# Chronic disease and smoking cessation intention: associations with oral health status, behaviors, and care in Korea

**DOI:** 10.3389/fpubh.2026.1742386

**Published:** 2026-02-13

**Authors:** Hye-Lim Hong, Nam-Hee Kim

**Affiliations:** 1 Department of Dental Hygiene, Graduate School, Yonsei University, Wonju, Republic of Korea; 2 Department of Dental Hygiene, Mirae Campus, Yonsei University, Wonju, Republic of Korea

**Keywords:** chronic disease, dental care, intention to quit, Korea Community Health Survey (KCHS), oral health, oral hygiene, smoking cessation

## Abstract

**Background:**

Oral conditions are immediately perceptible in daily life and may act as proximal factors associated with smokers' intention to quit, yet it is unclear whether these associations differ by chronic disease status.

**Methods:**

We analyzed nationally representative Korea Community Health Survey data (2010–2024) for current smokers aged ≥30 years (*N* = 522,377). The outcome was cessation intention (yes/no). Oral factors included self-rated oral health, masticatory discomfort, periodontal symptoms, oral-hygiene behaviors (post-lunch toothbrushing; dental flossing/interdental brushes), and dental service utilization (checkups, scaling, unmet dental care). Survey-weighted hierarchical logistic regression estimated associations in the total sample; models were repeated within strata defined by physician-diagnosed hypertension or diabetes. In the full sample, interaction terms (oral factor × chronic disease) were tested with Holm–Bonferroni adjustment.

**Results:**

Approximately 27% had chronic diseases; the survey-weighted prevalence of cessation intention was 62.6 vs. 66.0% in chronic-disease and non-chronic groups, respectively. After full adjustment, chronic disease was associated with higher odds of intention (OR = 1.19; 95% CI: 1.11–1.26). Oral status and oral-hygiene behaviors were positively associated with intention. Dental scaling remained positively associated (OR = 1.11; 95% CI: 1.04–1.17), whereas routine checkups were borderline/non-significant (OR = 1.06; 95% CI: 1.00–1.12). Reporting unmet dental care was also associated with higher intention (OR = 1.21; 95% CI: 1.14–1.29). No statistically significant heterogeneity across chronic-disease strata was observed; formal interaction tests were not significant after Holm–Bonferroni adjustment, and marginal predicted probabilities showed only small absolute differences.

**Conclusion:**

In this nationally representative sample, oral health status, hygiene behaviors, and dental care utilization showed modest yet consistent associations with smokers' intention to quit, without meaningful modification by chronic disease status. These findings support embedding evidence-based cessation support within routine dental and chronic-disease care and using observable oral health cues as conversation starters to motivate quitting.

## 1 Introduction

Smoking is a major risk factor for chronic diseases, including cancer and cardiovascular disease, and remains a leading cause of preventable mortality (1). Smoking cessation is the most effective public health strategy for reducing the burden of smoking-related diseases (2). Intention to quit is shaped by psychological and social contexts that influence subsequent quit attempts and success (3–7). Consistent with health behavior theories and cessation research, mere risk awareness is often insufficient to change behavior (8–11); identifying specific cues to action is critical (12–15).

Globally, about one in five adults uses tobacco—approximately 1.25 billion people in 2022—despite long-term declines since 2000, underscoring a persistent exposure relevant to oral and systemic health (16). WHO tracks smoking at country and regional levels via the Global Health Observatory and the STEPS NCD risk-factor surveillance platform (17, 18). Consistent with this framing, WHO's 2024 clinical guideline recommends integrating brief behavioral support and effective pharmacotherapies into routine care for adults who use tobacco (19), and the Global Oral Health Action Plan (2023–2030) identifies tobacco as a common risk factor to be addressed within integrated oral-health and primary-care services (20).

Within this context, oral health problems—such as tooth discoloration, halitosis, and oral pain—can make the harms of smoking immediately perceptible in daily life. It is hypothesized that these problems may function as proximal factors associated with readiness to quit by heightening perceived vulnerability, consistent with the health belief and transtheoretical models (8, 10, 11, 21–23). However, responsiveness to such cues may depend on a smoker's broader health context, particularly the presence of chronic disease.

Among individuals diagnosed with chronic diseases (e.g., hypertension or diabetes), intention formation may follow distinct psychosocial pathways (4, 24, 25). Prior literature suggests two plausible mechanisms: a health-crisis perspective, in which diagnosis heightens perceived vulnerability and renders additional problems—including oral symptoms—salient cues that may increase quit intention (6, 26, 27); and a desensitization/self-regulatory perspective, in which ongoing disease management can blunt responses to common warnings and subjective symptoms (28–31), whereas established health routines (e.g., toothbrushing, flossing) may align with intention formation as described in the transtheoretical model (4, 13, 14, 24).

Chronic disease status extends beyond diagnosis to shape health behaviors and identity and reflects sustained engagement with healthcare systems (32, 33). Accordingly, responses to oral problems—and their associations with cessation intention—may differ by chronic disease status. However, most previous studies have treated smokers as a homogeneous group, yielding limited evidence on whether oral health factors relate to quit intention differently within the context of chronic disease (34, 35).

Therefore, using nationally representative data from the Korea Community Health Survey, we aimed to examine whether oral health factors—including oral health status, oral hygiene behaviors, and dental service utilization—are associated with intention to quit smoking among current smokers, and whether these associations differ between smokers with and without chronic diseases (hypertension or diabetes). Our primary objective was to quantify these associations in the total sample and within strata defined by chronic disease status; our secondary objective was to assess whether any observed differences across strata indicate meaningful heterogeneity. We focused on hypertension and diabetes because these common, actively managed conditions are central to primary-care pathways where cessation support is routinely delivered (19, 20).

## 2 Materials and methods

### 2.1 Study design and participants

**Figure 1:**
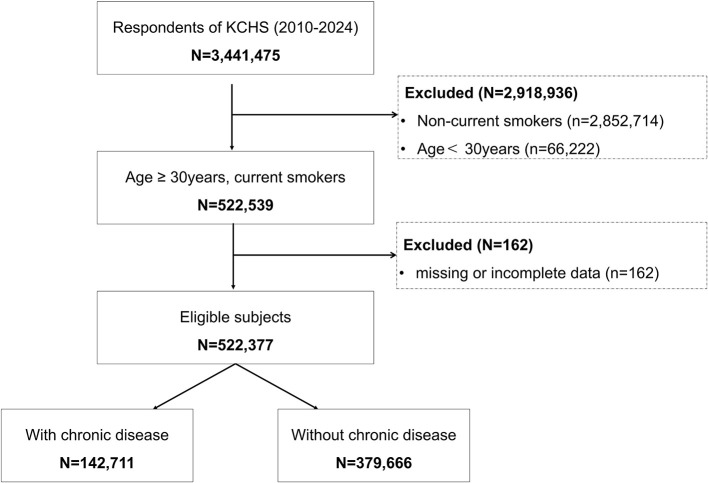
Flow diagram of sample selection from the Korea Community Health Survey (KCHS), 2010–2024; chronic disease was defined as physician-diagnosed hypertension or diabetes. Counts are unweighted.

We conducted a cross-sectional analysis of the Korea Community Health Survey (KCHS) 2010–2024, a nationally representative survey with complex sampling administered by the Korea Disease Control and Prevention Agency (KDCA) (36). From 3,441,475 respondents, we identified current smokers aged ≥30 years as defined in the KCHS instrument (*N* = 522,539). We excluded participants with missing smoking cessation intention or chronic disease status (*N* = 162), yielding a final analytic sample of *N* = 522,377. The flow of sample selection is summarized in Figure 1. Analyses were performed in the total sample and stratified by chronic disease status (hypertension or diabetes).

This study was a secondary analysis of de-identified, publicly available Korea Community Health Survey (KCHS) data. The Institutional Review Board (IRB) of Yonsei University waived ethical approval (Approval No. 1041849-202412-SB-266-01). The Korea Disease Control and Prevention Agency (KDCA) obtains informed consent and removes personal identifiers prior to public release.

### 2.2 Dependent variable

The outcome was smoking cessation intention, motivated by the transtheoretical model, which emphasizes intention as a proximal predictor of quitting (37). Responses to “Do you plan to quit smoking?” were categorized as intention to quit (plans to quit within 1 month, within 6 months, or someday) vs. no intention (no plans).

### 2.3 Independent variables

Guided by the health belief model (21), oral health factors covered three domains:

- Oral health status: self-rated oral health (good vs. poor), masticatory discomfort (yes vs. no), and periodontal symptoms (yes vs. no). Periodontal symptoms were coded “yes” if any of the following were reported: tooth mobility, heavy dental calculus, gum bleeding, or gum swelling; otherwise “no.” This composite indicator reflects how general periodontal symptoms are captured in the KCHS. These measures are based on self-reported symptoms rather than clinical examinations.
- Oral health behaviors: tooth brushing after lunch the previous day (yes vs. no) and regular use of dental floss or interdental brushes (yes vs. no).
- Oral health service utilization: dental checkups in the past year (yes vs. no), dental scaling in the past year (yes vs. no), and unmet dental care in the past year (yes vs. no).

For regression models, reference categories were: poor self-rated oral health, no masticatory discomfort, no periodontal symptoms, no tooth brushing after lunch, no flossing/interdental-brush use, no dental checkups, no scaling, and no unmet dental care (ref = No).

### 2.4 Stratification variable

Chronic disease status was defined as a self-reported physician diagnosis of hypertension or diabetes. These conditions were selected for their high prevalence, consistent measurement across all KCHS years, and centrality to primary care pathways where cessation support is routinely delivered (19, 20).

### 2.5 Control variables

Covariates included sociodemographic characteristics (age, gender, income, education, occupation), smoking related variables (age at smoking initiation, history of quit attempts), and health behaviors/status (alcohol consumption, walking activity, self-rated general health, stress, and depression).

### 2.6 Statistical analysis

We summarized characteristics using survey weighted descriptive statistics and chi square tests. We then estimated five nested, survey-weighted logistic regression models for cessation intention: Model 1 unadjusted; Model 2 additionally adjusted for oral health status; Model 3 added oral hygiene behaviors; Model 4 added oral health service utilization; Model 5 fully adjusted for all covariates.

To assess effect modification by chronic disease status, two complementary analyses were conducted: (a) stratified analyses re estimating Model 5 within chronic disease strata (no interaction terms) and comparing adjusted odds ratios (and 95% CIs) across strata; and (b) formal interaction testing in the full sample by adding oral factor × chronic disease terms to Model 5 and summarizing each as a ratio of odds ratios (ROR = OR_chronic/OR_non chronic) with design based Wald tests. Because eight interactions were examined, Holm–Bonferroni–adjusted *p* values are reported. For interpretability, marginal predicted probabilities by stratum were estimated from Model 5 using marginal standardization (covariates at observed values).

To address the 15-year pooling, we (i) included survey year (2010–2024) as a categorical covariate in all multivariable models (Models 2–5) to account for secular trends; and (ii) specified the complex survey design using KDCA-provided strata and primary sampling units with person-level sampling weights. When pooling years, weights were re-normalized within each survey year to have a mean of 1.0 before concatenation, to prevent certain survey years from disproportionately influencing pooled estimates due to differences in annual weight scaling. Design-based standard errors were computed via Taylor series linearization. All measures (exposures and outcome) were self-reported within the KCHS, and the cross-sectional design precludes inference about temporality or causality.

We used complete case analysis (*N* excluded = 162); because some items were not asked every year and some values were missing, denominators vary by variable as shown in the tables. Two-sided *p* < 0.05 was considered statistically significant. Analyses were conducted in R 4.5.1 (R Foundation for Statistical Computing, Vienna, Austria) using KCHS sampling weights and design-based variance estimation.

## 3 Results

Sample characteristics are summarized in Table 1; approximately 27% of participants had chronic diseases. The survey-weighted prevalence of cessation intention was 62.6% in the chronic disease group and 66.0% in the non-chronic group. Table 2 presents distributions among those reporting intention to quit, stratified by chronic disease status.

**Table 1:** Distribution of oral health–related characteristics by smoking cessation intention and chronic disease status among current smokers (*N* = 522,377).

**Characteristics**		**Total**		**Intention to quit**			**With chronic disease**			**Without chronic disease**		
		***N***	**%**	**Yes**	**No**	***P*-value**	**Yes**	**No**	***P*-value**	**Yes**	**No**	***P*-value**
Total, *N* (% of sample)		522,377	100	321,775 (65.2)	200,602 (34.8)		84,337 (62.6)	58,274 (37.4)		237,438 (66.0)	142,228 (34.0)	
**Oral health status**												
Self-rated oral health	Good	292,962	60.4	61.8	57.6	< 0.001	48.1	52.7	< 0.001	64.8	61.2	< 0.001
	Poor	229,385	39.6	38.2	42.4		51.9	47.3		35.2	38.8	
Masticatory discomfort	No	323,265	66.5	67.9	63.9	< 0.001	57.3	53.0	< 0.001	71.1	67.6	< 0.001
	Yes	199,076	33.5	32.1	36.1		42.7	47.0		28.9	32.4	
Periodontal symptoms	No	98,283	64.9	64.5	65.6	0.002	60.3	61.2	0.242	65.5	66.8	< 0.001
	Yes	50,820	35.1	35.5	34.4		39.7	38.8		34.5	33.2	
**Oral health behaviors**												
Tooth brushing	Yes	238,221	49.4	51.5	45.3	< 0.001	48.1	42.2	< 0.001	52.5	46.4	< 0.001
	No	284,031	50.6	48.5	54.7		51.9	57.8		47.5	53.6	
Dental flossing	Yes	12,858	19.1	21.5	14.3	< 0.001	19.5	11.5	< 0.001	22.0	15.0	< 0.001
	No	71,195	80.9	78.5	85.7		80.5	88.5		78.0	85.0	
**Oral health service utilization**												
Dental checkups	Yes	109,449	36.7	39.0	31.8	< 0.001	38.4	31.1	< 0.001	39.2	32.0	< 0.001
	No	245,872	63.3	61.0	68.2		61.6	68.9		60.8	68.0	
Dental scaling	Yes	107,045	36.1	38.4	31.3	< 0.001	38.5	30.5	< 0.001	38.3	31.6	< 0.001
	No	236,769	63.9	61.6	68.7		61.5	69.5		61.7	68.4	
Unmet dental care	Yes	105,498	22.6	23.8	20.5	< 0.001	23.3	20.5	< 0.001	23.9	20.5	< 0.001
	No	372,599	77.4	76.2	79.5		76.7	79.5		76.1	79.5	

Chronic disease status defined by self-reported physician-diagnosed hypertension or diabetes.

Counts are unweighted and percentages are survey-weighted.

Denominators vary by item due to missingness and item availability across survey years.

Group differences were tested using chi-square tests; *p*-values are two-sided (significance at *p* < 0.05).

**Table 2:** Distribution of oral health–related characteristics among current smokers with intention to quit, stratified by chronic disease status (*N* = 321,775).

**Characteristics**		**Total**		**With intention to quit**		
		***N***	**%**	**With chronic disease**	**Without chronic disease**	***P*-value**
Total, *N* (% of sample)		321,775	100	84,337 (23.0)	237,438 (77.0)	
**Oral health status**						
Self-rated oral health	Good	134,686	61.8	51.9	64.8	< 0.001
	Poor	187,077	38.2	48.1	35.2	
Masticatory discomfort	No	206,019	67.9	57.3	71.1	< 0.001
	Yes	115,733	32.1	42.7	28.9	
Periodontal symptoms	No	62,789	64.5	60.3	65.5	< 0.001
	Yes	33,137	35.5	39.7	34.5	
**Oral health behaviors**						
Tooth brushing	Yes	155,545	48.5	48.1	52.5	< 0.001
	No	166,166	51.5	51.9	47.5	
Dental flossing	Yes	9,449	21.5	19.5	22.0	< 0.001
	No	42,434	78.5	80.5	78.0	
**Oral health service utilization**						
Dental checkups	Yes	76,111	39.0	38.4	39.2	0.017
	No	148,314	61.0	61.6	60.8	
Dental scaling	Yes	74,819	38.4	38.5	38.3	0.711
	No	143,541	61.6	61.5	61.7	
Unmet dental care	Yes	69,024	23.8	23.3	23.9	0.004
	No	227,110	76.2	76.7	76.1	

Analysis restricted to current smokers reporting intention to quit smoking.

Chronic disease status defined as self-reported physician-diagnosed hypertension or diabetes.

Denominators vary by item due to missingness and item availability across survey years.

Group differences were tested using chi-square tests; *p*-values are two-sided (*p* < 0.05).

In survey-weighted logistic regression, chronic disease was associated with lower cessation intention in the unadjusted model (Model 1 OR = 0.86; 95% CI: 0.85–0.88). After sequential adjustment for oral factors, service use, sociodemographic and behavioral covariates, and survey year (Model 5), individuals with chronic diseases showed modestly higher odds of intending to quit (OR = 1.19; 95% CI: 1.11–1.26; Table 3).

**Table 3:** Hierarchical logistic regression analysis of associations between oral health–related factors and smoking cessation intention among current smokers (*N* = 522,377).

**Characteristics**	**Total (*****N*** = **522,377)**														
	**Model 1**			**Model 2**			**Model 3**			**Model 4**			**Model 5**		
	**OR**	**95% CI**		**OR**	**95% CI**		**OR**	**95% CI**		**OR**	**95% CI**		**OR**	**95% CI**	
		**LL**	**UL**		**LL**	**UL**		**LL**	**UL**		**LL**	**UL**		**LL**	**UL**
**Diagnosis of chronic disease (ref** = **no)**															
Yes	0.86^***^	0.85	0.88	1.17^***^	1.11	1.23	1.19^***^	1.12	1.27	1.11^***^	1.07	1.16	1.19^***^	1.11	1.26
**Oral health status**															
**Self-rated oral health (ref** = **poor)**															
Good	1.19^***^	1.17	1.21	1.10^***^	1.04	1.16							1.13^***^	1.06	1.20
**Masticatory discomfort (ref** = **no)**															
Yes	0.84^***^	0.82	0.85	1.11^***^	1.05	1.17							1.10^**^	1.03	1.17
**Periodontal symptoms (ref** = **no)**															
Yes	1.05^**^	1.02	1.08	1.07^**^	1.02	1.12							1.04	0.98	1.10
**Oral health behaviors**															
**Tooth brushing (ref** = **no)**															
Yes	1.28^***^	1.26	1.30				1.18^***^	1.13	1.25				1.18^***^	1.12	1.24
**Dental flossing (ref** = **no)**															
Yes	1.65^***^	1.56	1.74				1.26^***^	1.18	1.35				1.23^***^	1.15	1.31
**Oral health service utilization**															
**Dental checkups (ref** = **no)**															
Yes	1.38^***^	1.35	1.40							1.11^***^	1.08	1.15	1.06	1.00	1.12
**Dental scaling (ref** = **no)**															
Yes	1.36^***^	1.34	1.39							1.14^***^	1.10	1.18	1.11^**^	1.04	1.17
**Unmet dental care (ref** = **no)**															
Yes	1.21^***^	1.17	1.25							1.21^***^	1.17	1.25	1.21^***^	1.14	1.29

All models are survey-weighted.

Model 1: Unadjusted.

Model 2: Model 1 + oral health status (self-rated oral health, masticatory discomfort, periodontal symptoms).

Model 3: Model 2 + oral hygiene behaviors (post-lunch toothbrushing, dental flossing including interdental brushes).

Model 4: Model 3 + oral health service utilization (dental checkups, dental scaling, unmet dental care).

Model 5 (fully adjusted): Model 4 + sociodemographic (age, gender, education, income, occupation), smoking-related (age at initiation, previous quit attempts), health-related (alcohol use, walking activity, self-rated general health, stress, depression), and survey year (categorical, 2010–2024).

Reference categories: poor self-rated oral health; no masticatory discomfort; no periodontal symptoms; no post-lunch toothbrushing; no dental flossing (incl. interdental brush); no routine dental checkups; no dental scaling; unmet dental care = No.

Notation: OR, odds ratio; CI, confidence interval; LL, lower limit; UL, upper limit. Two-sided tests; ^**^p < 0.01, ^***^p < 0.001.

Across oral factors in the total sample (Model 5), good self-rated oral health (vs. poor) and oral hygiene behaviors (post lunch toothbrushing; flossing/interdental brush use) were positively associated with cessation intention. Dental scaling remained positively associated, whereas routine checkups did not after full adjustment (checkups OR = 1.06; 95% CI: 1.00–1.12; scaling OR = 1.11; 95% CI: 1.04–1.17; Table 3). Reporting unmet dental care (Yes vs. No) was also positively associated overall (OR = 1.21; 95% CI: 1.14–1.29).

**Figure 2:**
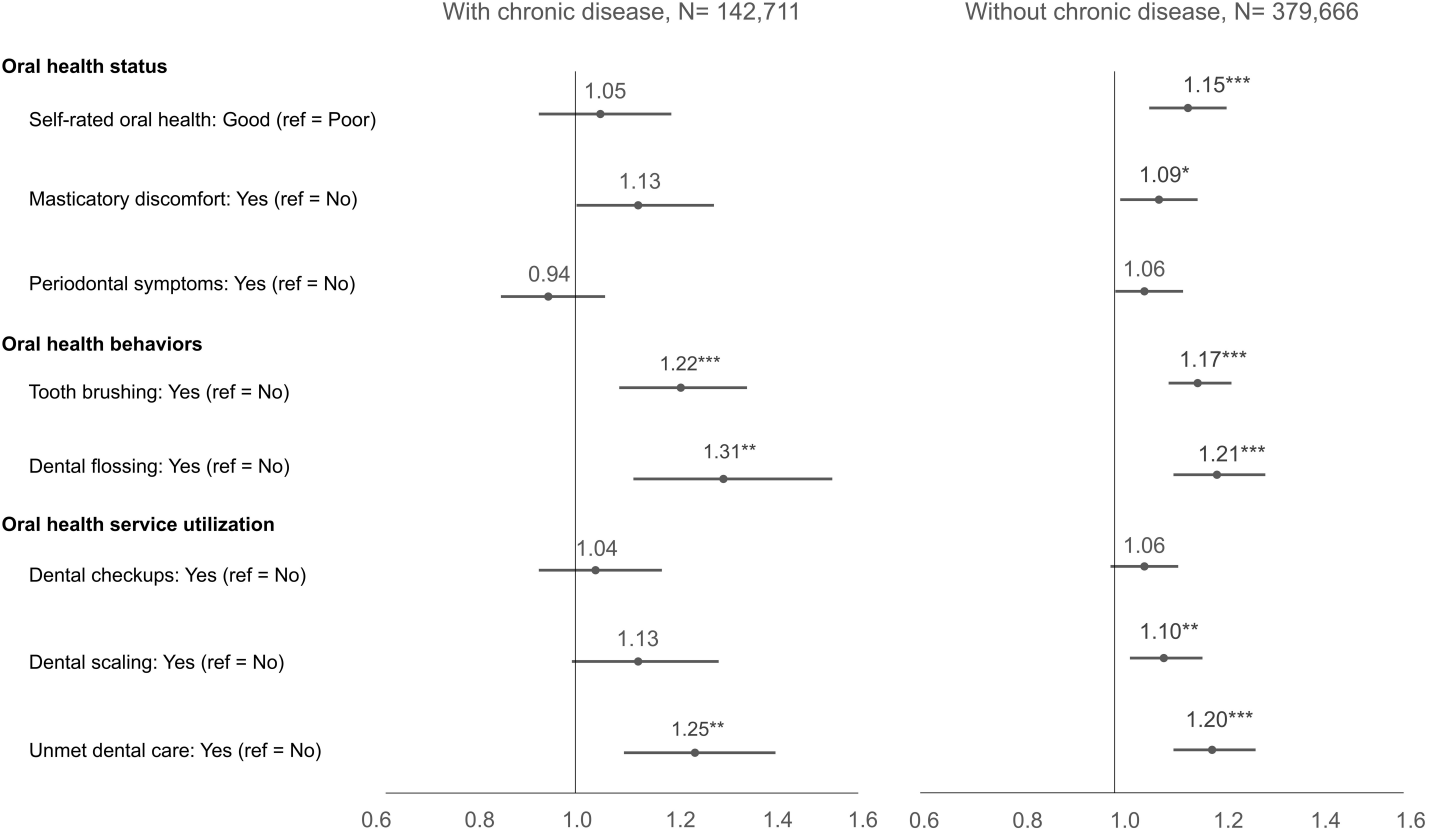
Forest plot of adjusted associations between oral factors and intention to quit smoking, stratified by chronic-disease status. Points denote adjusted odds ratios with 95% CIs from survey-weighted logistic regression (Model 5); the vertical line indicates OR = 1. Interaction *p*-values (Holm–Bonferroni adjusted) are provided in Supplementary Table S3 and were not statistically significant. Two-sided tests; *p < 0.05, **p < 0.01, ***p < 0.001.

Stratified models yielded directionally consistent associations across chronic disease strata, and point estimates differed modestly with overlapping confidence intervals (Supplementary Tables S1, S2; Figure 2). Reporting unmet dental care was positively associated with cessation intention in both strata (Supplementary Tables S1, S2; Figure 2).

In fully adjusted models, no oral factor × chronic disease interaction reached statistical significance after Holm–Bonferroni correction. Ratios of odds ratios were near 1.0 with 95% CIs spanning unity (Supplementary Table S3), and marginal predicted probabilities showed only small absolute differences between strata (Supplementary Table S4). These results emphasize the modest effect sizes and their consistency across groups rather than statistical interaction.

## 4 Discussion

Using nationally representative KCHS data, we assessed whether associations between oral health factors and smoking cessation intention differ by chronic disease status. After sequential adjustment for oral factors, service use, sociodemographic and behavioral covariates, and survey year, smokers with chronic diseases had modestly higher odds of intending to quit (Model 5). Across oral factors, post-lunch toothbrushing and flossing were positively associated with intention in the total sample and within strata, while dental scaling remained positively associated after full adjustment and routine checkups did not (Table 3; Figure 2). These findings indicate that tangible oral symptoms and treatment-related improvements (e.g., reduced bleeding or perceived freshness after scaling) may serve as cues to action that motivate smoking cessation intention more strongly than routine checkups (8, 21). This contrast suggests that active, symptom-related dental care may be more closely tied to motivational readiness than passive or informational service use. Formal interaction tests were non-significant after Holm–Bonferroni correction; ratios of odds ratios were near 1.0, and marginal predicted probabilities showed only small absolute between-group differences (Supplementary Tables S3, S4), indicating no statistical evidence of effect modification by chronic disease status.

The crude negative association for chronic disease (Model 1) that became positive after full adjustment (Model 5) is most plausibly due to confounding—for example, by age, health behaviors, and healthcare contact—rather than a true “reversal.” Although compatible with a “teachable moment” interpretation in which illness experiences and care encounters elevate quitting motivation (38, 39), the cross-sectional design means temporality and causality cannot be inferred; smokers already intending to quit may be more likely to engage with care. Our interpretation therefore emphasizes associations rather than causal effects.

A notable pattern was the positive association between unmet dental care and quit intention (Model 5), observed in both strata (Supplementary Tables S1, S2; Figure 2). One explanation is that unresolved symptoms (e.g., pain, halitosis, aesthetic concerns) function as salient proximal cues that heighten perceived vulnerability and prompt intention, consistent with the health belief and transtheoretical models (8, 21, 24). An alternative—and not mutually exclusive—account is behavioral clustering: individuals with greater health orientation (reflected in hygiene routines) report stronger quit intention, while those with unmet needs experience symptom salience despite access barriers. Our data cannot adjudicate between these pathways; both remain plausible and actionable.

In this context, reverse causality should also be considered. In our data, individuals with an intention to quit smoking exhibited more favorable oral health indicators and higher use of some preventive services (e.g., dental scaling), raising the possibility that pre-existing quitting motivation or broader health orientation influenced oral health–related behaviors and perceptions. Nevertheless, prior studies have shown that oral health complaints, such as dental pain or tooth sensitivity, can serve as salient cues that prompt cessation intention by increasing symptom salience and perceived risk (22, 23), which is consistent with the direction of the associations observed in the present study.

Mechanisms consistent with prior literature support these interpretations. Visible or symptomatic oral conditions can operate as proximal cues that increase readiness to change (40, 41), and objective signals such as oral malodor have been linked to higher quit intention (42, 43). At the same time, repeated exposure to generic warnings may exhibit wear out (43, 44), and competing priorities in chronic disease could diffuse attention from common oral symptoms (28, 43, 44). Together with possible clustering of protective behaviors across adulthood (45, 46), these processes help explain the modest but consistent associations we observed.

These findings should be understood within the broader national, cultural, and social context of South Korea, where long-standing norms of regular dental visits and a universal national health insurance system with relatively high coverage for preventive dental services and chronic disease management may shape the meaning and salience of indicators such as dental scaling and unmet dental care. In settings with more limited primary oral healthcare or fragmented chronic disease management, these associations may differ, clarifying the boundary conditions and international relevance of the present findings.

Practice implications follow pragmatically and align with current guidance (19, 20): because interactions were not statistically significant, subgroup specific effect claims are unwarranted. A universal, care embedded approach is indicated. In dental settings, pair brief behavioral support with pharmacotherapy and leverage concrete, visible oral signs (discoloration, halitosis, discomfort) to personalize advice and link to assistance. In chronic disease care, integrate the same brief support into routine visits, using encounters as potential teachable moments while avoiding causal overreach. These recommendations are consistent with the pattern of scaling related associations and the non-significant checkup estimates in fully adjusted models (Table 3; Figure 2).

Strengths include the very large, nationally representative sample; pre-specified stratification by chronic disease status; and design-based estimation with survey-year indicators to address secular trends over 2010–2024. Limitations include the cross-sectional design; self-reported oral exposures and outcomes (potential misclassification and social desirability bias); the use of a composite self-reported periodontal symptom indicator that combines clinically heterogeneous conditions, potentially limiting specificity; possible residual confounding (e.g., nicotine dependence); and an operational definition of chronic disease restricted to hypertension and diabetes for measurement consistency, which does not capture smoking-related morbidity (e.g., COPD, cardiovascular disease, or cancer) or other clinically relevant conditions affecting oral health, potentially underestimating the broader impact of chronic disease status. Because intention is common, odds ratios may overstate relative risks; effect sizes were modest (~1.1–1.2) and statistical significance likely reflects the large *N*. To aid interpretation, we report marginal predicted probabilities (Supplementary Table S4). Finally, smoking cessation intention does not necessarily translate into actual cessation behavior; therefore, the present findings should be interpreted as associations with motivational readiness rather than realized behavior change. Although we adjusted for survey year and used design-based variance estimation, pooling 15 years cannot fully eliminate temporal shifts in instrumentation or response tendencies. In addition, sex- and age-stratified analyses were not pre-specified to avoid multiplicity and loss of power; chronic disease status was the *a priori* effect modifier of interest.

Future research should prospectively test whether perceptible oral cues (e.g., malodor, discoloration) predict transitions from intention to quit attempts and abstinence, and whether chairside interventions that combine behavioral support with pharmacotherapy outperform brief advice alone in both dental and chronic disease clinics (47, 48). Pragmatic trials could evaluate “scaling plus cessation” bundles and assistance uptake as intermediate endpoints (49).

In sum, oral status, hygiene behaviors, and selected service use indicators showed modest, positive, and consistent associations with quit intention, and these associations did not differ materially by chronic disease status after multiplicity adjusted testing, reinforcing a universal, care embedded strategy rather than subgroup specific approaches.

## 5 Conclusion

In this nationally representative sample of adult smokers, oral health status, hygiene behaviors, and selected dental service utilization demonstrated modest yet consistent associations with smokers' intention to quit. These associations were not significantly modified by the presence of chronic diseases. Collectively, these findings underscore the necessity of a comprehensive, integrated intervention strategy: embedding evidence-based smoking cessation support within both dental and chronic disease management settings. This should involve leveraging observable oral health indicators to customize patient communication and ensuring the systematic provision of cessation treatment or appropriate referrals during routine clinical encounters.

## Data Availability

Publicly available datasets were analyzed in this study. This data can be found here: the data that support the findings of this study are available from the Korea Disease Control and Prevention Agency's Korea Community Health Survey (KCHS) repository (https://chs.kdca.go.kr/chs/rawDta/rawDtaProvdMain.do). These data are publicly accessible to qualified researchers upon registration with the KDCA. Any derived analysis code or aggregated outputs are available from the corresponding author upon reasonable request.
