# Supplementary material for: Chronic disease and smoking cessation intention: associations with oral health status, behaviors, and care in Korea
Source: Front Public Health. 2026 Feb 13;14:1742386. doi: 10.3389/fpubh.2026.1742386 (PMC12945789; doi:10.3389/fpubh.2026.1742386)
Supplement: Supplementary file 1 [file Table_1.docx]

Supplementary Table S1**.** Hierarchical logistic regression analysis of associations between oral health factors and smoking cessation intention among current smokers with chronic diseases (N=142,711)

|  | **Case: With chronic disease (N = 142,711)** | | | | | | | | | | | | | | |
| --- | --- | --- | --- | --- | --- | --- | --- | --- | --- | --- | --- | --- | --- | --- | --- |
|  | **Model 1** | | | **Model 2** | | | **Model 3** | | | **Model 4** | | | **Model 5** | | |
|  | **OR** | **95% CI** | | **OR** | **95% CI** | | **OR** | **95% CI** | | **OR** | **95% CI** | | **OR** | **95% CI** | |
|  |  | **LL** | **UL** |  | **LL** | **UL** |  | **LL** | **UL** |  | **LL** | **UL** |  | **LL** | **UL** |
| Self-rated oral health (ref = Poor) |  |  |  |  |  |  |  |  |  |  |  |  |  |  |  |
| Good | 1.20*** | 1.06 | 1.26 | 1.05 | 0.94 | 1.17 |  |  |  |  |  |  | 1.05 | 0.92 | 1.20 |
| Masticatory discomfort (ref = No) |  |  |  |  |  |  |  |  |  |  |  |  |  |  |  |
| Yes | 0.84*** | 0.81 | 0.86 | 1.13* | 1.02 | 1.26 |  |  |  |  |  |  | 1.13 | 1.00 | 1.29 |
| Periodontal symptoms (ref = No) |  |  |  |  |  |  |  |  |  |  |  |  |  |  |  |
| Yes | 1.04 | 0.98 | 1.10 | 1.01 | 0.91 | 1.11 |  |  |  |  |  |  | 0.94 | 0.84 | 1.06 |
| Tooth brushing (ref = No) |  |  |  |  |  |  |  |  |  |  |  |  |  |  |  |
| Yes | 1.26*** | 1.23 | 1.30 |  |  |  | 1.24*** | 1.11 | 1.38 |  |  |  | 1.22*** | 1.09 | 1.36 |
| Dental flossing (ref = No) |  |  |  |  |  |  |  |  |  |  |  |  |  |  |  |
| Yes | 1.87*** | 1.65 | 2.12 |  |  |  | 1.35*** | 1.16 | 1.58 |  |  |  | 1.31** | 1.12 | 1.54 |
| Dental checkups (ref = No) |  |  |  |  |  |  |  |  |  |  |  |  |  |  |  |
| Yes | 1.38*** | 1.33 | 1.44 |  |  |  |  |  |  | 1.06 | 0.99 | 1.14 | 1.04 | 0.92 | 1.18 |
| Dental scaling (ref = No) |  |  |  |  |  |  |  |  |  |  |  |  |  |  |  |
| Yes | 1.43*** | 1.37 | 1.49 |  |  |  |  |  |  | 1.15*** | 1.07 | 1.24 | 1.13 | 0.99 | 1.30 |
| Unmet dental care (ref = No) |  |  |  |  |  |  |  |  |  |  |  |  |  |  |  |
| Yes | 1.18*** | 1.13 | 1.22 |  |  |  |  |  |  | 1.16*** | 1.07 | 1.24 | 1.25** | 1.10 | 1.42 |

Adjustment variables identical to Table 3.

Note: OR = odds ratio; CI = confidence interval; LL = lower limit; UL = upper limit.
Two-sided tests; * p<0.05, ** p<0.01, *** p<0.001.

Supplementary Table S2**.** Hierarchical logistic regression analysis of associations between oral health factors and smoking cessation intention among current smokers without chronic diseases (N=379,666)

|  | **Control: Without chronic disease (N = 379,666)** | | | | | | | | | | | | | | |
| --- | --- | --- | --- | --- | --- | --- | --- | --- | --- | --- | --- | --- | --- | --- | --- |
|  | **Model 1** | | | **Model 2** | | | **Model 3** | | | **Model 4** | | | **Model 5** | | |
|  | **OR** | **95% CI** | | **OR** | **95% CI** | | **OR** | **95% CI** | | **OR** | **95% CI** | | **OR** | **95% CI** | |
|  |  | **LL** | **UL** |  | **LL** | **UL** |  | **LL** | **UL** |  | **LL** | **UL** |  | **LL** | **UL** |
| Self-rated oral health (ref = Poor) |  |  |  |  |  |  |  |  |  |  |  |  |  |  |  |
| Good | 1.17*** | 1.15 | 1.19 | 1.11*** | 1.05 | 1.18 |  |  |  |  |  |  | 1.15*** | 1.07 | 1.23 |
| Masticatory discomfort (ref = No) |  |  |  |  |  |  |  |  |  |  |  |  |  |  |  |
| Yes | 0.85*** | 0.84 | 0.87 | 1.10** | 1.04 | 1.17 |  |  |  |  |  |  | 1.09* | 1.01 | 1.17 |
| Periodontal symptoms (ref = No) |  |  |  |  |  |  |  |  |  |  |  |  |  |  |  |
| Yes | 1.06** | 1.02 | 1.10 | 1.09** | 1.03 | 1.15 |  |  |  |  |  |  | 1.06 | 1.00 | 1.14 |
| Tooth brushing (ref = No) |  |  |  |  |  |  |  |  |  |  |  |  |  |  |  |
| Yes | 1.28*** | 1.26 | 1.30 |  |  |  | 1.17*** | 1.11 | 1.24 |  |  |  | 1.17*** | 1.11 | 1.24 |
| Dental flossing (ref = No) |  |  |  |  |  |  |  |  |  |  |  |  |  |  |  |
| Yes | 1.60*** | 1.51 | 1.70 |  |  |  | 1.25*** | 1.16 | 1.34 |  |  |  | 1.21*** | 1.12 | 1.31 |
| Dental checkups (ref = No) |  |  |  |  |  |  |  |  |  |  |  |  |  |  |  |
| Yes | 1.37*** | 1.34 | 1.40 |  |  |  |  |  |  | 1.13*** | 1.08 | 1.17 | 1.06 | 0.99 | 1.13 |
| Dental scaling (ref = No) |  |  |  |  |  |  |  |  |  |  |  |  |  |  |  |
| Yes | 1.35*** | 1.31 | 1.38 |  |  |  |  |  |  | 1.14*** | 1.10 | 1.19 | 1.10** | 1.03 | 1.18 |
| Unmet dental care (ref = No) |  |  |  |  |  |  |  |  |  |  |  |  |  |  |  |
| Yes | 1.22*** | 1.20 | 1.25 |  |  |  |  |  |  | 1.22*** | 1.18 | 1.27 | 1.20*** | 1.12 | 1.29 |

Adjustment variables identical to Table 3.

Note: OR = odds ratio; CI = confidence interval; LL = lower limit; UL = upper limit.
Two-sided tests; * p<0.05, ** p<0.01, *** p<0.001.

Supplementary Table S3. Interaction between chronic disease status and oral factors for intention to quit smoking: survey-weighted logistic regression, KCHS 2010–2024 (Model 5)

| **Oral factor (reference)** | **OR non-chronic**  **(95% CI)** | **OR chronic**  **(95% CI)** | **ROR**  **(95% CI)** | **p_interaction** | **p_adj (Holm)** | **N Without chronic** | **N With chronic** |
| --- | --- | --- | --- | --- | --- | --- | --- |
| Self-rated oral health (ref = Poor) |  |  |  |  |  |  |  |
| Good | 1.15 (1.07–1.23) | 1.05 (0.92–1.20) | 0.91 (0.79–1.05) | .182 | >.999 | 379,645 | 142,702 |
| Masticatory discomfort (ref = No) |  |  |  |  |  |  |  |
| Yes | 1.09 (1.01–1.17) | 1.13 (1.00–1.29) | 1.05 (0.91–1.21) | .512 | >.999 | 379,636 | 142,705 |
| Periodontal symptoms (ref = No) |  |  |  |  |  |  |  |
| Yes | 1.06 (1.00–1.14) | 0.94 (0.84–1.06) | 0.90 (0.78–1.03) | .114 | .912 | 113,872 | 35,231 |
| Tooth brushing (ref = No) |  |  |  |  |  |  |  |
| Yes | 1.17 (1.11–1.24) | 1.22 (1.09–1.36) | 1.02 (0.90–1.15) | .748 | >.999 | 379,572 | 142,680 |
| Dental flossing (ref = No) |  |  |  |  |  |  |  |
| Yes | 1.21 (1.12–1.31) | 1.31 (1.12–1.54) | 1.08 (0.91–1.29) | .374 | >.999 | 64,848 | 19,205 |
| Dental checkups (ref = No) |  |  |  |  |  |  |  |
| Yes | 1.06 (0.99–1.13) | 1.04 (0.92–1.18) | 0.95 (0.83–1.10) | .497 | >.999 | 265,816 | 89,505 |
| Dental scaling (ref = No) |  |  |  |  |  |  |  |
| Yes | 1.10 (1.03–1.18) | 1.13 (0.99–1.30) | 1.03 (0.89–1.20) | .708 | >.999 | 259,104 | 84,710 |
| Unmet dental care (ref = No) |  |  |  |  |  |  |  |
| Yes | 1.20 (1.12–1.29) | 1.25 (1.10–1.42) | 1.04 (0.90–1.20) | .630 | >.999 | 344,866 | 133,231 |

Survey-weighted logistic regression with the same covariates and survey design as Model 5.

Interaction p from design-based Wald tests; p_adj by Holm–Bonferroni across eight interactions (α family-wise=0.05).

Note: OR = odds ratio; CI = confidence interval; ROR = OR_chronic / OR_non-chronic.

Reference categories: poor self-rated oral health; no masticatory discomfort; no periodontal symptoms; no post-lunch toothbrushing; no dental flossing (including interdental brush); no regular dental checkups; no dental scaling; unmet dental care = no.

Percentages are weighted, counts are unweighted.

Supplementary Table S4. Marginal predicted probabilities by chronic disease status and oral factors (Model 5)

| **Oral factor** | **Group** | **X level** | **Predicted probability (%)** | **95% CI** | **Difference (Yes − No) pp (%p)** | **95% CI** | **P value** |
| --- | --- | --- | --- | --- | --- | --- | --- |
| Self-rated oral health | Without chronic | Good | 34.0 | 30.2–37.9 | 2.6 | -2.7–7.9 | 0.331 |
|  |  | Poor | 31.4 | 27.7–35.1 |  |  |  |
|  | With chronic | Good | 38.0 | 34.0–41.9 | 2.8 | -2.7–8.2 | 0.322 |
|  |  | Poor | 35.2 | 31.4–39.0 |  |  |  |
| Masticatory discomfort | Without chronic | No | 31.4 | 27.7–35.1 | 2.0 | -3.2–7.2 | 0.450 |
|  |  | Yes | 33.4 | 29.8–37.1 |  |  |  |
|  | With chronic | No | 35.2 | 31.4–39.0 | 2.1 | -3.2–7.4 | 0.440 |
|  |  | Yes | 37.3 | 33.5–41.0 |  |  |  |
| Periodontal symptoms | Without chronic | No | 31.4 | 27.7–35.1 | 0.8 | -4.5–6.0 | 0.770 |
|  |  | Yes | 32.2 | 28.4–36.0 |  |  |  |
|  | With chronic | No | 35.1 | 31.4–39.0 | 0.9 | -4.6–6.3 | 0.766 |
|  |  | Yes | 36.1 | 32.0–39.9 |  |  |  |
| Tooth brushing | Without chronic | Yes | 35.1 | 31.1–39.0 | 3.7 | -1.7–9.1 | 0.185 |
|  |  | No | 31.4 | 27.7–35.1 |  |  |  |
|  | With chronic | Yes | 39.0 | 35.0–43.1 | 3.8 | -1.7–9.4 | 0.177 |
|  |  | No | 35.2 | 31.4–39.0 |  |  |  |
| Dental flossing | Without chronic | Yes | 36.0 | 31.7–40.2 | 4.6 | -1.1–10.2 | 0.114 |
|  |  | No | 31.4 | 27.7–35.1 |  |  |  |
|  | With chronic | Yes | 40.0 | 35.6–44.3 | 4.8 | -1.0–10.6 | 0.107 |
|  |  | No | 35.2 | 31.4–39.0 |  |  |  |
| Dental checkups | Without chronic | Yes | 32.5 | 28.6–36.5 | 1.1 | -4.3–6.5 | 0.682 |
|  |  | No | 31.4 | 27.7–35.1 |  |  |  |
|  | With chronic | Yes | 36.4 | 32.3–40.4 | 1.2 | -4.4–6.8 | 0.676 |
|  |  | No | 35.2 | 31.4–39.0 |  |  |  |
| Dental scaling | Without chronic | Yes | 33.6 | 29.5–37.6 | 2.2 | -3.3–7.6 | 0.439 |
|  |  | No | 31.4 | 27.7–35.1 |  |  |  |
|  | With chronic | Yes | 37.5 | 33.3–41.6 | 2.3 | -3.4–7.9 | 0.430 |
|  |  | No | 35.2 | 31.4–39.0 |  |  |  |
| Unmet dental care | Without chronic | Yes | 35.7 | 31.6–39.7 | 4.3 | -1.2–9.7 | .128 |
|  |  | No | 31.4 | 27.7–35.1 |  |  |  |
|  | With chronic | Yes | 39.7 | 35.5–43.8 | 4.5 | -1.1–10.1 | .120 |
|  |  | No | 35.2 | 31.4–39.0 |  |  |  |

Predicted probabilities from emmeans(type="response") on Model 5; averages are marginal over covariates.

Difference is Yes minus No within chronic group, in percentage points (pp).
